# Evaluation of Fracture Resistance of Occlusal Veneers Made of Different Types of Materials Depending on Their Thickness

**DOI:** 10.3390/ma16176006

**Published:** 2023-08-31

**Authors:** Łukasz Czechowski, Beata Dejak, Bartłomiej Konieczny, Michał Krasowski

**Affiliations:** 1 Department of Prosthodontics MU of Łódź, 92-213 Łódź, Poland; 2 University Laboratory of Materials Research, Medical University of Łódź, 92-213 Łódź, Poland

**Keywords:** fracture resistance, occlusal veneers, dental ceramics

## Abstract

Pathological tooth wear is an escalating social problem. Occlusal veneers can be an alternative to traditional prosthetic restorations such as crowns, inlays, and onlays. Background: The aim of this study is to assess the fracture resistance of occlusal veneers made of various materials depending on their thickness. Methods: In total, 120 occlusal veneers were examined. The restorations were made of four ceramics: leucite LC (IPS Empress Esthetic), hybrid HC (Vita Enamic), lithium disilicate LDC (IPS e.max Press), and zirconium oxide ZOC (Ceramill Zolid HT). A total of 30 veneers were made of each material, 10 for each of the three thicknesses: 1 mm, 1.5 mm, 2 mm. The restorations were cemented on identical abutments duplicated from the developed phantom tooth 35 (KaVo) with composite cement (All Bond Universal). The samples prepared in this way were subjected to a compressive strength test in a universal testing machine. Statistical analysis of the results was performed. Results: The average fracture resistance of occlusal veneers made of zirconium oxide ceramic was 1086–1640 N, of lithium disilicate ceramics 456–1044 N, of hybrid ceramics 449–576 N, and of leucite ceramics 257–499 N. Conclusions: Occlusal veneers made of ceramics, zirconium oxide and lithium disilicate, had the highest resistance to fractures. Restorations made of leucite ceramics turned out to be the least resistant to forces. The greater the thickness of the ceramic occlusal veneers, the greater their fracture resistance.

## 1. Introduction

Pathological tooth wear is a common problem in society [1]. As a result of attrition, erosion and abrasion, a loss of hard tooth tissues on the occlusal surfaces occurs [2]. So far, onlays, overlays, and crowns have been used to restore teeth, unfortunately these were associated with a significant additional loss of hard tooth tissues. Occlusal veneers are a less invasive restoration. They are characterized by covering only the occlusal surface of the tooth, they do not require the preparation of axial walls or its central part. The most commonly used materials presented in the literature from which these restorations are made are lithium disilicate ceramics, zirconium oxide reinforced lithium silicate, zirconium oxide ceramics as well as new generations of ceramics such as hybrid ceramics and nanoceramics [3].

Leucite ceramics (LC) are a modification of feldspar ceramics. They consist of tetragonal leucite crystals embedded in a glassy, amorphous mass of silica [4]. They are characterized by having aesthetics, color, and transparency similar to enamel [5,6,7]. Lithium disilicate ceramics (LDC) consist of needle-shaped lithium disilicate crystals 0.5 μm wide and 4 μm long, and lithium orthophosphate, embedded in silica. The refractive index of the described ceramics is similar to the enamel, which makes it possible to achieve good optical properties [4,5,6]. Lithium silicate ceramics (ZORLSC) are composed of lithium silicate crystals with the addition of zirconium dioxide, which constitutes 10–11% of their mass. Crystals with a size of 0.5–0.7 μm are embedded in the silica matrix [7,8]. Zirconium dioxide ceramics (ZOC) 3Y-TZP consist of more than 99% densely sintered zirconium dioxide crystals. The allotropic tetragonal variety of these ceramics requires yttrium oxide stabilization at room temperature. Compared to glass ceramics, 3Y-TZP ceramics are more opaque [9]. Their variants are 4Y-PSZ and 5Y-PSZ ceramics, which contain a greater amount of optically isotropic cubic phase, which results in greater translucency [7,9,10]. A new solution on the market of dental materials are hybrid ceramics (HC) (polymer infiltrated ceramic network, PICN, e.g., Vita Enamic (Vita)). Their skeleton is formed by a ceramic mesh (86% by weight) consisting of silica (58–63%) and aluminum oxide (20–23%). This matrix is impregnated with UDMA resin, TEGDMA (14% by weight) [7,11,12,13]. Another new material is nanoceramics (NC), an example of which is Lava Ultimate (3M ESPE). The main component of this material is a nanofiller made of silanized silica with a diameter of 20 nm and zirconium with a diameter of 4 to 11 nm. The crystals are grouped into nanoclusters and embedded in resin [4,11,12]. The basic mechanical parameters of the above-mentioned materials are summarized in Table 1.

So far, research has not yielded clear answers as to which material is optimal for making occlusal veneers. Schlichting et al. observed CAD–CAM ceramics (e.max CAD) and composite (Lava Ultimate) ultra-thin CAM occlusal veneers for 3 years. They showed statistically comparable efficacy of both restorations according to USPHS criteria, although greater surface degradation was found in the group of composite resins [14]. According to fatigue studies by Mueller et al. the indirect resin composite groups showed better fatigue behavior compared to lithium disilicate [15]. Heck et al. showed that composite occlusal veneers (Lava Ultimate) could prove more durable than restorations made of leucite ceramics (IPS Empress CAD) and showed similar resistance as lithium disilicate restorations (IPS e.max CAD) [16]. According to Comba et al. studies, PICN (Vita Enamic) occlusal veneers had smaller fracture resistance than veneers made of lithium disilicate ceramic (IPS e.max CAD) [17].

It is assumed that restorations (onlays, overlays, crowns) made of conventional ceramics and composites should have a minimum thickness on the occlusal surface of the posterior teeth of 1.5–2 mm on working cusps and 1–1.5 mm on non-working ones [18]. Occlusal veneers make it possible to reduce the necessary tooth preparation in relation to traditional restorations. Their thickness depends on the material of which they are made and the conditions in the patient’s mouth and the planned treatment. The thickness of occlusal veneers presented in the literature varies depending on the material in the range: 0.3–1.5 mm for leucite ceramics, 0.3–1.5 mm for hybrid ceramics, 0.3–2 mm for lithium disilicate ceramics, and 0.5–1.5 mm for zirconium oxide ceramics. Valenzuela et al., Johnson et al., and Egbert et al. suggest the possibility of using such restorations made of hybrid ceramics or lithium disilicate with a thickness of 0.3 mm [12,19,20]. Essam et al. suggest that 0.5 mm thick ceramic occlusal veneers of lithium disilicate can be successfully used for lateral tooth veneers [21]. A similar thickness of restorations is suggested by Zahran et al. for zirconium oxide reinforced lithium silicate ceramic restorations [22]. Sasse et al. recommend keeping the thickness above 0.7 mm [23]. Although 0.5 mm thick zirconium oxide ceramic restorations had a fracture resistance far exceeding the maximum forces acting in the mouth, due to the difficulty of their execution, the authors who studied them, Ioanidis et al. and Maeder et al., suggest increasing their thickness to about 1 mm [3,24]. According to some authors, the increase in the thickness of occlusal veneers had a significant impact on their fracture resistance, not all researchers achieved similar results. In their research, Valeznuela et al. showed statistically significantly higher fracture resistance for lithium disilicate ceramic veneers with a thickness of 0.3 mm than for those with a thickness of 0.6 mm [19]. The thickness of this type of restoration is ultimately dictated by the clinical situation. As a minimally invasive restoration, it aims at the smallest possible reduction in the remaining hard tissues of the tooth. In some situations, however, occlusal veneers with a thickness of up to 2 mm may be required. Fracture resistance increases with the thickness of veneers, but as the researchers point out, this also leads to a statistically significant increase in stresses in the restorations themselves, while their value decreases in the cement layer [25]. The choice of material is also important for the stresses arising in the restorations. The larger the elastic modulus of the material, the greater the stresses arising in it while the values of stress in the cement layer are lower [25,26].

Occlusal veneers are fairly new type of restoration with probable disadvantages like decemntation, fractures, chipping and cracks as a result of certain mechanical and thermal loads. Previous in vitro studies assessing the impact of the type of material and the thickness of occlusal veneers on their fracture resistance have not given conclusive answers. 

The aim of the study was to assess the fracture resistance of occlusal veneers made of various types of materials depending on their thickness.

The null hypothesis was that load bearing capacity would not be significantly different between occlusal veneers made of different materials and between different thicknesses of those restorations.

## 2. Materials and Methods

### 2.1. Preparation of the Abutment

A total of 120 identical artificial abutments of first left lower premolar teeth were used for the study. The occlusal surface of lower, left, second premolar 35 (KaVo Dental, Biberah, Germany), was prepared for the occlusal veneer, preserving the natural inclination of the cusp slopes of 120° [27]. The developed tooth was positioned centrally in Express XT Putty Soft silicone compound (3M ESPE, Saint Paul, MI, USA) in a cubic form made in the M200 digital 3D printer (Zortrax, Olsztyn, Poland). Its long axis was parallel to the vertical. The whole element was duplicated in Picodent Twinsil silicone mass (Picodent, Wipperfürth, Germany). In this way, the negatives of tooth sample 35 were created. Silicone dies were used to make 120 identical abutments from Vertex Self Curing acrylic material (Vertex-Dental B. V., Soesterberg, The Netherlands)).

### 2.2. Preparation of Restorations

**Figure 1:**
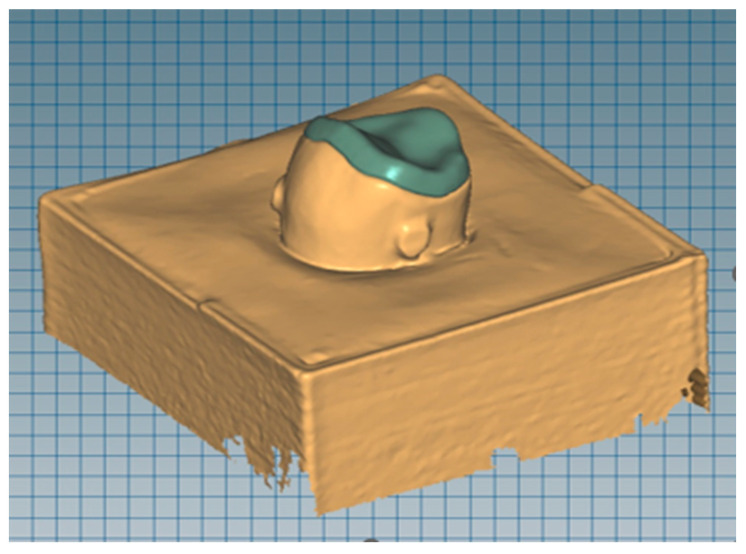
Scanned acrylic abutment with designed occlusal veneer.

The first abutment was digitized using the Map 300 digital 3D scanner (Amann Girbach AG, Koblach, Austria). Then, with the help of Ceramill Mind (Amann Girbach AG, Koblach, Austria), designs for occlusal veneers with thicknesses of 1, 1.5, and 2 mm were prepared (Figure 1). Their thickness was uniform in every part of the restoration. Four different materials were used for the restoration: leucite ceramics, lithium disilicate ceramics, hybrid ceramics, and zirconium oxide ceramics. A total of 30 veneers were made of each material, 10 for each of the three thicknesses tested. Restorations made of leucite ceramic IPS Empress Esthetic (Ivoclar Vivadent, Schaan, Lichtenstein) and lithium disilicate IPS e.max Press (Ivoclar Vivadent, Schaan, Lichtenstein) were made by pressing. The designed restorations were milled in Ceramill Wax (Amann Girbach AG, Koblach, Austria) in a Ceramill Motion milling machine (Amann Girbach AG, Koblach, Austria). The wax patterns were embedded in molds in the refractory mass Bellavest SH (Bego, Bremen, Germany) + BegoSol HE (Bego, Bremen, Germany). Then, in a Programat Ep 3000 (Ivoclar Vivadent, Schaan, Lichtenstein) oven, wax was fired, and ceramic material was pressed. Veneers made of transparent zirconium oxide 4Y-TPZ Ceramill Zolid HT (Amann Girbach AG, Koblach, Austria) and Vita Enamic (Vita Zhanfabrik, Bad Säckigen, Germany) hybrid ceramics were made by milling. The designed reconstructions were milled from prefabricated blocks in a Ceramill Motion (Amann Girbach AG, Koblach, Austria) milling machine in a 1:1 scale.

### 2.3. Luting

Before cementation, the chewing surfaces of all abutments were sandblasted with 50 μm grain diameter alumina under a pressure of 3.5 bar. Then the All Bond Universal (Bisco, Schaumburg, IL, USA) adhesive was applied to their surface in accordance with the manufacturer’s recommendations. Two separate layers of bond were rubbed in for 10–15 s and then blown with a blower. After application, the bond was exposed to the light of a curing lamp for 10 s.

Restoration surfaces made of leucite ceramics were etched with 9% hydrofluoric acid for 60 s (Table 2). Lithium disilicate and hybrid ceramic restorations were etched with 4.5% hydrofluoric acid for 20 and 60 s, respectively. Then two layers of Porcelain Primer silane (Bisco, Schaumburg, IL, USA) were applied to the etched surfaces, left for 30 s, and gently blown out. Subsequently, one layer of the All Bond Universal (Bisco, Schaumburg, IL, USA) bonding system was applied and blown. The restoration surfaces prepared in this way were treated with the light of a curing lamp for 10 s in accordance with the manufacturer’s recommendations.

Zirconium oxide ceramic restorations were sandblasted with aluminum oxide with a grain diameter of 50 μm at a pressure of 3.5 bar. Two layers of Z-PRIME Plus (Bisco, Schaumburg, IL, USA) zirconium oxide ceramic primer were applied to the restorations.

To cement all occlusal veneers, Duo-Link Universal (Bisco, Schaumburg, IL, USA) composite dual-bonding cement was used. The material was applied to the surface of the abutments, and the restorations. Next the restorations were positioned on the abutments. Excess cement was removed, and then the edges of the fillings were light cured for 2–3 s on each side [28]. After stabilizing the restorations on the abutments, they were additionally light cured for 40 s on each side (Table 2). The samples prepared in this way were placed for 24 h in a water bath at 37 °C.

### 2.4. Compressive Strength Test

**Figure 2:**
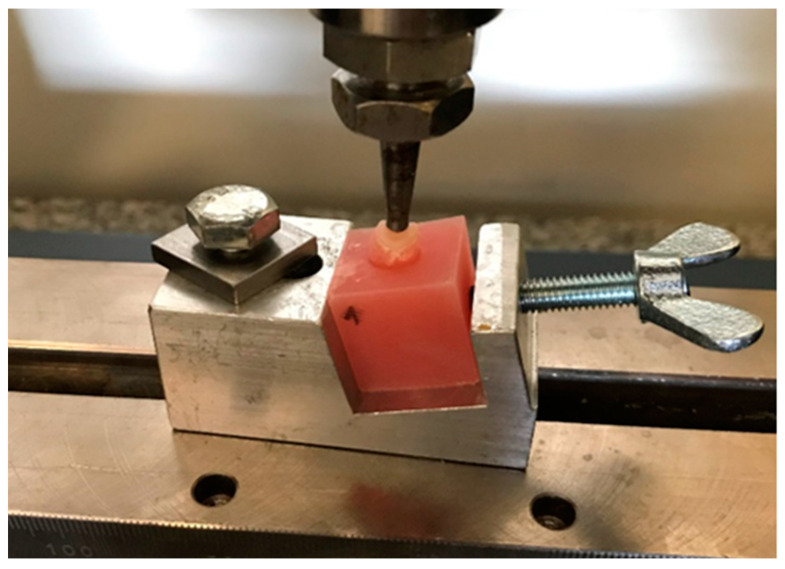
Sample placed in a universal testing machine.

The study was carried out in the universal testing machine Z020 (Zwick/Roell, Ulm, Germany) at the University Material Research Laboratory of the Medical University of Łódź. The samples were placed in a specially prepared holder providing forces on the sample at an angle of 15° BL [27]. The mutual angle of the premolar teeth axis LB is 15 degrees: of the premolars of the maxilla (6 degrees) and mandible (9 degrees). Therefore, the tooth was positioned relative to the head at this angle to simulate loading conditions similar to those in the mouth. The pressure was exerted by a metal head ending with a ball with a diameter of 3.5 mm. The diameter of the metal ball of the head is due to the width of the functional cusps of the opposing teeth (Figure 2). The head speed has been set at 1 mm/min. The results of the study were recorded in the form of graphs of the force acting on the samples depending on the displacement of the head (N). The test was recorded, and the moment of fracture of the sample on the recording was compared with a graph in order to read the value of the destructive force of the sample.

### 2.5. Statistical Analysis

For statistical analysis of the results, Microsoft Excel from Microsoft Office 2010 and Statistica v. 13 were used. The following statistical parameters were evaluated: arithmetic mean, median—as average measures, as well as standard deviation. The minimum and maximum values were also given. To assess the distribution of individual parameters, the Shapiro–Wolf normality test was used. In case of a distribution inconsistent with the normal distribution, the Kruskall–Wallis test was used. In the situation of a normal distribution of individual parameters, the equality of variance was assessed using the Levene test. For equal variances, the ANOVA test was used with the Scheffe post-hoc test. The assumed significance level was α = 0.05.

## 3. Results

**Figure 3:**
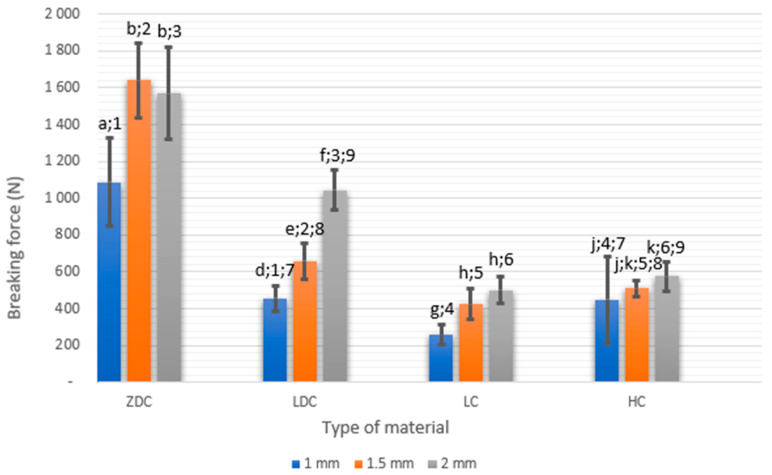
Comparison of fracture resistance (N) of occlusal veneers depending on the material used. Bars with different letters and digits have different statistical significance (*p* < 0.05). Letters are for comparison of same material with different thicknesses, digits are for comparison of same thicknesses between different materials.

The average values of the fracture resistance of occlusal veneer samples depending on the material used are shown in Figure 3. The highest resistance was obtained by restorations made of zirconium oxide ceramics 1086–1640 N. The fracture resistance of reconstructions made of lithium disilicate ceramics was 456–1044 N. Much lower values of fracture resistance were obtained by occlusal veneers made of hybrid ceramics 449–576 N, while restorations made of leucite ceramics fractured under the force of 257–499 N. 

Occlusal veneers made of leucite ceramics had the lowest fracture resistance. Occlusal restorations made of disilicate ceramics and hybrid ceramics had similar fracture resistance at thicknesses of 1 and 1.5 mm. Veneers made of lithium disilicate ceramics proved to be a favorable material for occlusal reconstructions. Occlusal veneers made of zirconium oxide ceramics were the most resistant to fracture of all the studied groups, regardless of their thickness. They were damaged during operation of 2–3 times higher loads than other restorations made of other materials.

The thickness of the occlusal veneers had a significant impact on their fracture resistance. Restorations made of leucite, hybrid, lithium disilicate, and zirconium oxide ceramics with a thickness of 1 mm had average fracture resistances of 257 ± 52.6 N, 449 ± 236.2 N 456 ± 67.79 N, 1086 ± 239.75 N, respectively; restorations with a thickness of 1.5 mm 424 ± 82. N, 509 ± 42.35 N, 658 ± 99.52 N, 1640 ± 200.33 N, respectively; and veneers with a thickness of 2 mm 499.89 ± 73.98 N, 576.6 ± 80.63 N, 1044.4 ± 111.20 N, and 1569 ± 252.34 N, respectively. With increasing thickness, the fracture resistance of restorations increased in all material groups, with the exception of occlusal veneers made of zirconium oxide ceramics with a thickness of 1.5 and 2 mm. 

The weakest in terms of mechanics turned out to be veneers with a thickness of 1 mm made of leucite ceramics. They were damaged already under the influence of an average force of 257 N. They were almost 2 times less resistant than 1 mm hybrid and lithium disilicate ceramic restorations and over 4 times less resistant than zirconium oxide ceramic restorations. Veneers 1 mm thick made of lithium disilicate ceramic and hybrid ceramics had similar fracture resistance (456.1, 449.7 N). Restorations 1 mm thick made of zirconium oxide ceramics turned out to be the most resistant to forces. They broke under the influence of an average force of 1086 N comparable to veneers made of lithium disilicate with a thickness of 2 mm 1044 N. 

Leucite ceramic restorations with a thickness of 2 mm had 94% greater fracture resistance (499.9 N) than 1 mm. Lithium disilicate ceramic veneers with a thickness of 2 mm had a fracture resistance 58% higher than that of 1.5 mm thick and 128% more than 1 mm thick. Occlusal restorations made of hybrid ceramics with a thickness of 2 mm and 1.5 mm were broken by a similar force. Reconstructions from zirconium oxide ceramics with a thickness of 2 mm showed fracture resistance 44% higher than those with a thickness of 1 mm. On the other hand, ZrO2 occlusal veneers with thicknesses of 1.5 and 2 mm were damaged under a similar load.

**Figure 4:**
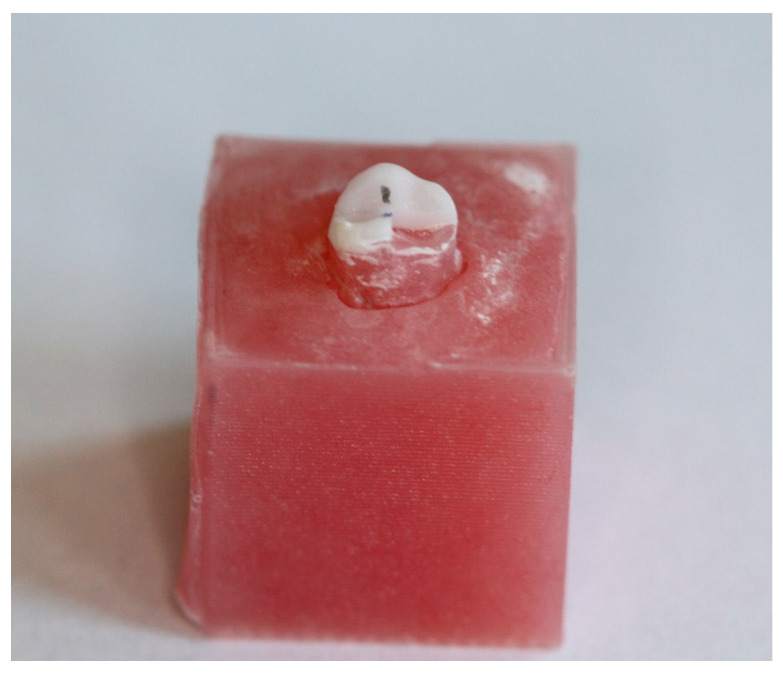
Sample after testing.

All samples of leucite and hybrid ceramics, were defragmented during testing. Lithium disilicate ceramic restorations also mostly defragmented except for four samples with a thickness of 1.5 mm and seven with a thickness of 2 mm. Zirconium oxide ceramic samples mostly broke into two parts or had a single fragment brake off from them (Figure 4).

Statistically significant differences between the tested materials and thicknesses are presented in Table 3.

## 4. Discussion

The null hypothesis was rejected. Both the type of material from which the occlusal veneers were made, and their thickness had a significant impact on their fracture resistance. Occlusal veneers made of leucite ceramics had the lowest fracture resistance. Occlusal restorations made of disilicate ceramics and hybrid ceramics with thicknesses of 1 mm and 1.5 mm were damaged under similar forces. Veneers made of lithium disilicate ceramics proved to be a beneficial material for occlusal plane reconstructions, especially when the restorations were 2 mm thick. Occlusal veneers made of zirconium oxide ceramics were the most resistant to fracture of all the studied groups, regardless of their thickness.

The fracture resistance of veneers made of the presented materials correlated to a large extent with their mechanical properties [5,7,29,30]. Veneers made of materials with higher flexural strength had greater fracture resistance. Zirconium oxide ceramics are characterized by the highest flexural strength among other dental ceramics amounting to 900–1200 MPa [5,13]. Their high strength is significantly influenced by their construction. They are a polycrystalline and a polymorphic material. This occurs in three allotropic forms: monocyclic, tetragonal, and cylindrical. In order to preserve the tetragonal structure at room temperature, this material must be stabilized, for example, with yttrium oxide [7]. In the area of the crack formed under the influence of external forces, the grains are transformed again from the tetragonal form to the monocyclic form [5]. In the course of this process, there is an increase in the volume of material by about 3–5%. This increase leads to the closure of the gap. The increase in volume as a result of this transformation is called the strengthening transformation. It prevents toughness fractures of zirconium oxide ceramics and significantly increases the fracture resistance of this material [7,31].

Lithium disilicate ceramics have a flexural strength of 330–400 MPa, three times lower than that of zirconium oxide ceramics [5,13]. This material consists of 60% lithium disilicate crystals, embedded in a glassy matrix. The large number of these longitudinal, irregularly arranged crystals are responsible for the mechanical properties of this ceramic. A crack initiated in silica is blocked on numerous lithium disilicate crystals, which prevents its further propagation [5,31,32].

Leucite ceramics are composed of tetragonal leucite crystals (20–55%) embedded in a glassy amorphous silica mass [4,32]. The addition of these crystals causes an increase in mechanical resistance. Microcracks appearing in the material that encounter crystals change their direction, which causes the loss of some energy [4,5]. The flexural strength of this ceramic is about 100 MPa.

Hybrid ceramics combine the features of ceramic and composite materials. They consist of two intertwined networks connected chemically [7]. The majority of their mass (about 75% of the material volume) is a glass ceramic network penetrated with a methacrylate resin network (25% of the material volume). Thanks to this combination, hybrid ceramics are a more rigid material than composites, while being more flexible than other ceramics. Their Young’s modulus is 37 GPa and their flexural strength is about 150 MPa [13]. A crack progressing in the ceramic material is dispersed as it passes through its resin component [13]. For this reason, hybrid ceramics show better fracture resistance than leucite ceramics. 

Studies by other authors confirm our results. Al-Akhali et al., Andrade et al. and Albelasy et al. showed that lithium disilicate ceramic restorations had greater fracture resistance than those made of hybrid ceramics [33,34,35,36]. In the research of Ioannidis et al. and Maeder et al., zirconium oxide ceramic restorations had a fracture resistance greater than lithium disilicate and hybrid ceramic restorations [3,24]. 

In the current study, the average fracture resistance of veneers made of various ceramics was almost twice lower than in the studies of other authors. According to Ionidas et al., Maeder et al., and Al-Zordk et al., the fracture resistance of veneers made of lithium disilicate ceramics with a thickness of 1 mm was 1110–2505 N, while in this work it was equal to 456 N [3,24,37]. Hybrid ceramic veneers in the literature had a fracture resistance of 891–2505 N, and in this dissertation this value reached an average of 449 N [3,24,35,37]. In the literature, zirconium oxide ceramic restorations with a thickness of 1 mm had an average fracture resistance of 1779–2256 N, while in the conducted study this was 1086 N [3,24,37]. Lower values of fracture resistance obtained in own research may be caused by the use of PMMA as a foundation material. The elastic modulus of the acrylic from which the foundation is made of is low (1.2–2.2 GPa) [38]. In studies by other authors, occlusal veneers were cemented to tooth tissues that are more rigid—enamel 80 GPa and dentin 19 GPa [38,39]. The combination of occlusal veneers with rigid tooth structures provides them with greater resistance to fractures. In addition, the strength of the bond of the resin cement with the hard tissues of teeth is much stronger than with acrylic material, and the correct complex combining reconstruction with the tooth significantly improves the fracture resistance of the restoration [3]. 

In this study only static forces were applied. No aging methods were used. It is important to note that fracture resistance test should be complemented by a thermomechanical fatigue resistance test to better understand the long-term clinical aspects of choosing the right material for occlusal veneers. Also, the influence of the material chosen for restoration on the opposing teeth is an important factor. Baldi et al. simulated a bruxism scenario in molars restored with occlusal veneers and healthy teeth. They showed that the material type had significant influence on the wear of the restorations and antagonist teeth. While composite based indirect restorations (PINC) had higher wear than lithium silicate restorations, they also had smaller wear in the enamel of antagonist teeth [40]. Despite these limitations, in our study, the resistance of occlusal veneers made of zirconium oxide ceramics (regardless of thickness) and lithium disilicate ceramics (with a thickness of 1.5 and 2 mm) significantly exceeded the maximum chewing forces in the mouth. According to Singh et al., these are about 486 N for women and 606 N for men [41]. Similarly, according to the research of de Abreu et al., 420 N for women and 630 N for men [42]. 

The thickness of the occlusal veneers also had a significant impact on their fracture resistance. Veneers with a thickness of 1 mm turned out to be the least resistant to fractures. Veneers 1 mm thick made of leucite ceramics were particularly vulnerable to damage, and were destroyed already with an average force of 257 N. Increasing the thickness of these veneers to 1.5–2 mm resulted in a twofold increase in fracture resistance (499.9 N). Similarly, a twofold increase in fracture resistance was observed between lithium disilicate veneers with a thickness of 1–1.5 mm and 2 mm (456.1 N and 1044.4 N). Hybrid ceramic veneers had similar resistance to fractures regardless of thickness. The highest resistance to damage reaching 1640 N was characterized by veneers with a thickness of 1.5–2 mm made of zirconium oxide ceramics, although veneers made of this material with a thickness of 1 mm also broke when exposed to a high force of 1086 N. Zirconium oxide ceramic veneers with a thickness of 1 mm had comparable fracture resistance to two times thicker veneers made of lithium disilicate ceramic.

As the thickness increased, the resistance increased regardless of the material used. The research of most authors confirms this relationship. The thicker the restorations on the chewing surface, the more resistant to fracture they are [3,24,35,36]. The thickness of occlusal veneers made of zirconium oxide ceramics can be limited to 1 mm. Hybrid ceramic and lithium disilicate veneers should be at least 1.5 mm thick on the occlusal surface to withstand the forces occurring in the mouth. For strength reasons, it is not recommended to make occlusal veneers from leucite ceramics, and if they were to be used, their thickness should be 2 mm.

Occlusal veneers made of zirconium oxide ceramic are characterized by the highest fracture resistance. Unfortunately, zirconium oxide ceramics are one of the hardest and most wear-resistant ceramics. This is a smooth material, so it does not significantly affect the abrasion of opposing teeth, but its adaptive abrasion in contact with opposing teeth is very low, which can lead to occlusion disorders.

Lithium disilicate ceramic occlusal veneers are recommended for occlusal reconstruction. Their hardness is not much greater than the hardness of enamel, thanks to which it does not cause attrition of the opposing teeth. The material can be bonded adhesively to tissues of the teeth.

Leucite ceramics are a very fragile and not very durable material. Occlusal veneers made of them are the least resistant to fractures and they should not be used to rebuild the occlusal surfaces of teeth.

The search for materials for long-term restoration of occlusal surfaces of teeth is still ongoing. One of the newer solutions is hybrid ceramics. They have the advantages of ceramic and composite materials. Unfortunately, according to the conducted research, restorations made of this material are not very resistant to static forces.

## 5. Conclusions

1. Occlusal veneers made of zirconium oxide and lithium disilicate ceramics had the highest fracture resistance values. Restorations made of leucite ceramics turned out to be the least resistant to forces.
2. The greater the thickness of the ceramic occlusal veneers, the greater their fracture resistance. The thickness of occlusal veneers made of zirconium oxide ceramics can be limited to 1 mm. Hybrid ceramic and lithium disilicate veneers should be at least 1.5 mm thick on the occlusal surface to withstand the forces occurring in the mouth. For strength reasons, it is not recommended to make occlusal veneers from leucite ceramics, and if they are to be used, their thickness should be 2 mm.

## Figures and Tables

**Table 1:** Selected mechanical properties of ceramics used to make occlusal veneers.

	Leucite Ceramic (LC)	Lithium Disilicate Ceramic (LDC)	Zirconia Reinforced Lithium Silicate Ceramic (ZORLSC)	Zirconium Dioxide Ceramic (ZOC)	Hybrid Ceramic (HC)	Nanoceramic (NC)
Hardness (according to Vickers) (GPa)	6.6	5.3	6.5	13	2.5	2.6
Elastic modulus (GPa)	65–71	103	105	210	35–37	12
Flexural strength (MPa)	109–182	330–400	440	900–1200	150	160
Composition	tetragonal leucite crystals embedded in a glassy, amorphous mass of silica	needle-shaped lithium disilicate crystals 0.5 μm wide and 4 μm long, and lithium orthophosphate, embedded in silica	lithium silicate crystals with the addition of zirconium dioxide, which constitutes 10–11% of its mass and silica matrix	99% densely sintered zirconium dioxide crystals	ceramic mesh (86% by weight) consisting of silica (58–63%) and aluminum oxide (20–23%) impregnated with UDMA resin, TEGDMA (14% by weight)	silanized silica (diameter of 20 nm) and zirconium (diameter of 4 to 11 nm) crystals grouped in nanoclusters embedded in resin

**Table 2:** Luting process of occlusal veneers and abutments.

	Abutment Preparation		Occlusal Veneers Preparation		Cementation
LC	Chewing surfaces of all abutments were sandblasted with 50 μm grain diameter alumina under a pressure of 3.5 bar	Two separate layers of All Bond Universal (Bisco) were rubbed in for 10–15 s and then blown with a blower. After application, the bond was exposed to the light of a curing lamp for 10 s	Etched with 9% hydrofluoric acid for 60 s	Two layers of Porcelain Primer silane (Bisco) applied to the etched surfaces, left for 30 s and gently blown out. Subsequently, one layer of the All Bond Universal (Bisco) bonding system applied and blown. The restoration surfaces prepared in this way were treated with the light of a curing lamp for 10 s in accordance with the manufacturer’s recommendations	Duo-Link Universal (Bisco) was applied to the surface of the abutments and the restorations, light cured for 2–3 s on each side, excess removed, additionally light cured for 40 s on each side
LDC			Etched with 4.5% hydrofluoric acid for 20 s		
HC			Etched with 4.5% hydrofluoric acid for 60 s		
ZDC			Sandblasted with aluminum oxide with a grain diameter of 50 μm at a pressure of 3.5 bar	Two layers of Z-PRIME Plus (Bisco) zirconium oxide ceramic primer were applied to the restorations	

**Table 3:** Average values and standard deviations of destructive forces of occlusal veneers with statistical significance.

Material	1 mm	1.5 mm	2 mm	SIG.
	Mean ± SD	Mean ± SD	Mean ± SD	
Leucite ceramic (LC)	257.00 ± 52.60	424.30 ± 82.90	499.89 ± 73.89	*p* < 0.05; *p* = 0.00000
				1 mm < 1.5 mm; *p* = 0.00014
				1 mm < 2 mm; *p* = 0.00000
Hybrid Ceramic (HC)	449.70 ± 236.20	509.10 ± 42.35	576.60 ± 80.63	*p* < 0.05; *p* = 0.0273
				1 mm < 2 mm; *p* = 0.000558
Lithium disilicate ceramic (LDC)	456.10 ± 67.79	658.90 ± 99.52	1044.4 ± 111.20	*p* < 0.05; *p* = 0.00000
				1 mm < 1.5 mm; *p* = 0.00024
				1 mm < 2 mm; *p* = 0.00000
				1.5 < 2 mm; *p* = 0.00000
Zirconium dioxide ceramic (ZDC)	1086.1 ± 239.75	1640.0 ± 200.33	1569.0 ± 252.34	*p* < 0.05; *p* = 0.000002
				1 mm < 1.5 mm; *p* = 0.00006
				1 mm < 2 mm; *p* = 0.00035
SIG.	*p* < 0.05; *p* = 0.0001	*p* < 0.05; *p* = 0.0000	*p* < 0.05; *p* = 0.0000	
	ZDC > LC; *p* = 0.00000	ZDC > LC; *p* = 0.00001	ZDC > LC; *p* = 0.00001	
	ZDC > HC; *p* = 0.00415	ZDC > HC; *p* = 0.00035	ZDC > HC; *p* = 0.00010	
	LDC > LC; *p* = 0.01443	LDC > LC; *p* = 0.01506	LDC > LC; *p* = 0.00541	

## Data Availability

Data can be provided upon request from corresponding author.
